# Spontaneous Remission in Paroxysmal Nocturnal Hemoglobinuria—Return to Health or Transition Into Malignancy?

**DOI:** 10.3389/fimmu.2018.01749

**Published:** 2018-08-02

**Authors:** Eva-Stina Korkama, Anna-Elina Armstrong, Hanna Jarva, Seppo Meri

**Affiliations:** ^1^Immunobiology Research Program, Department of Bacteriology and Immunology, University of Helsinki, Helsinki, Finland; ^2^Coagulation Disorder Unit, Helsinki University Hospital Comprehensive Cancer Center, Helsinki, Finland; ^3^Helsinki University Hospital Laboratory (HUSLAB), Helsinki, Finland

**Keywords:** paroxysmal nocturnal hemoglobinuria, aplastic anemia, spontaneous remission, leukemia

## Abstract

Paroxysmal nocturnal hemoglobinuria (PNH) is an acquired syndrome characterized by intravascular hemolysis, thrombosis, and bone marrow failure. The disease is caused by a mutation in the *PIG-A* gene that leads to the lack of glycosylphosphatidylinositol-anchored complement regulatory molecules CD55 and CD59 on affected blood cell surfaces. In previous studies, spontaneous clinical remissions have been described. The disease manifestations are very heterogeneous, and we wanted to examine if true remissions and disappearance of the clone occur. In a follow-up of a nation-wide cohort of 106 Finnish patients with a PNH clone, we found six cases, where the clone disappeared or was clearly diminished. Two of the patients subsequently developed leukemia, while the other four are healthy and in clinical remission. According to our data, spontaneous remissions are not as frequent as described earlier. Since the disappearance of the PNH cell clone may indicate either a favorable or a poor outcome—remission or malignancy—careful clinical monitoring in PNH is mandatory. Nevertheless, true remissions occur, and further studies are needed to understand the immunological background of this phenomenon and to obtain a better understanding of the natural history of the disease.

## Introduction

Paroxysmal nocturnal hemoglobinuria (PNH) is an acquired syndrome characterized by intravascular hemolysis, thrombosis, and bone marrow (BM) failure (1). The disease is caused by a mutation in the *PIG-A* gene that leads to the lack of glycosylphosphatidylinositol (GPI)-anchored molecules, including the complement regulatory proteins CD55 and CD59 from the surface of a clonal lineage of blood cells (2–4). In addition, rare forms of PNH with mutations in the CD59 gene have been described indicating the key role of the absence of CD59 in the disease (5, 6).

The diagnosis of PNH is made by analyzing GPI-anchored molecules or the anchor itself on blood cells by flow cytometry. The disease course is often unpredictable, and the therapeutic options are limited. While in previous studies spontaneous remissions have been reported to occur in up to 15–30% of cases (7, 8), there are only few detailed case reports published on these patients. Recently, four cases of spontaneous remission were described in two different studies (9, 10), but doubts exist whether remissions really occur in PNH patients. In particular, it would be important to distinguish true remissions from other developing disorders, especially from malignancy, because in both cases the PNH diagnostics may give a negative result.

Because of the variable clinical course of PNH, we wanted to explore whether true spontaneous remissions occur in PNH in the Finnish patient material that we have collected during a long period of time. A nation-wide study of all PNH patients diagnosed in Finland since 1995 and an extended follow-up time (up to 20 years) have provided a unique opportunity to perform a detailed analysis of the course of the disease in our patients.

## Patients and Methods

In a nation-wide project, we collected patients from all Health Care Districts in Finland (with a total population of 5.6 million). The sources of information included the Helsinki University Central Hospital Laboratory (HUSLAB) databases, flow cytometry analysis of red blood cells (RBCs) and leukocytes, patients’ medical records, and a patient questionnaire. The patients were evaluated until September 2016, and the remission cases were followed until June 2017. In total, 106 patients with a CD59-deficient cell clone in flow cytometry were included in the study.

Besides the flow cytometry analysis the laboratory parameters included levels of hemoglobin (Hb), lactate dehydrogenase, haptoglobin, erythrocytes, leukocytes, platelets, inflammation markers (CRP, ESR), D-dimer, fibrinogen, coagulation factor VIII, creatinine, and ventricular natriuretic peptide. In addition, the glomerular filtration rates and BM analyses were performed. The clinical data included the symptoms and conditions attributable to PNH: hemoglobinuria, thrombotic events, infections, fatigue, renal failure, pulmonary hypertension, abdominal pain, dysphagia, dyspnea, erectile dysfunction, anemia, and the co-existence of PNH with aplastic anemia (AA), myelodysplastic syndrome (MDS), or leukemia.

An ethical permission for the study was provided by the coordinating ethical committee at the Helsinki University Hospital Health Care District. A research permission was also obtained from the National Institute of Health and Welfare (THL, Helsinki, Finland), and the patients gave written informed consents for the study.

## Results

In a cohort of 106 Finnish patients with a PNH clone, we found 6 cases, where the clone disappeared or clearly diminished (to a level equal or below 1.5%). Two of the six patients subsequently developed leukemia (acute myeloid leukemia and chronic myelomonocytic leukemia), while the other four are in clinical remission (Table 1).

**Table 1:** Patients and treatments.

	Age at PNH diagnosis (year)	Age at AA/BM failure diagnosis (year)	Treatment
Patient 1	23 years (1990)^a^	19 years (1986)	Androgens, corticosteroids
Patient 2	56 years (1998)	Hypoplastic BM 57 years (1999)	Androgens, corticosteroids
Patient 3	24 years (1998)	21 years (1995)	Antithymocyte globulin, prednisolone, cyclosporine, granulocyte growth factor
Patient 4	25 years (1998)	19 years (1992)	Cyclosporine, androgens
Patient 5	43 years (2000)	43 years (2000)	Antithymocyte globulin, prednisolone, cyclosporine
Patient 6	44 years (1998)	43 years (1997)	Prednisolone, cyclosporine

*PNH, paroxysmal nocturnal hemoglobinuria; AA, aplastic anemia; BM, bone marrow*.

*^a^Diagnosis by Ham’s test. First flow cytometry in 2001. For further clinical information, please see the text*.

**Figure 1:**
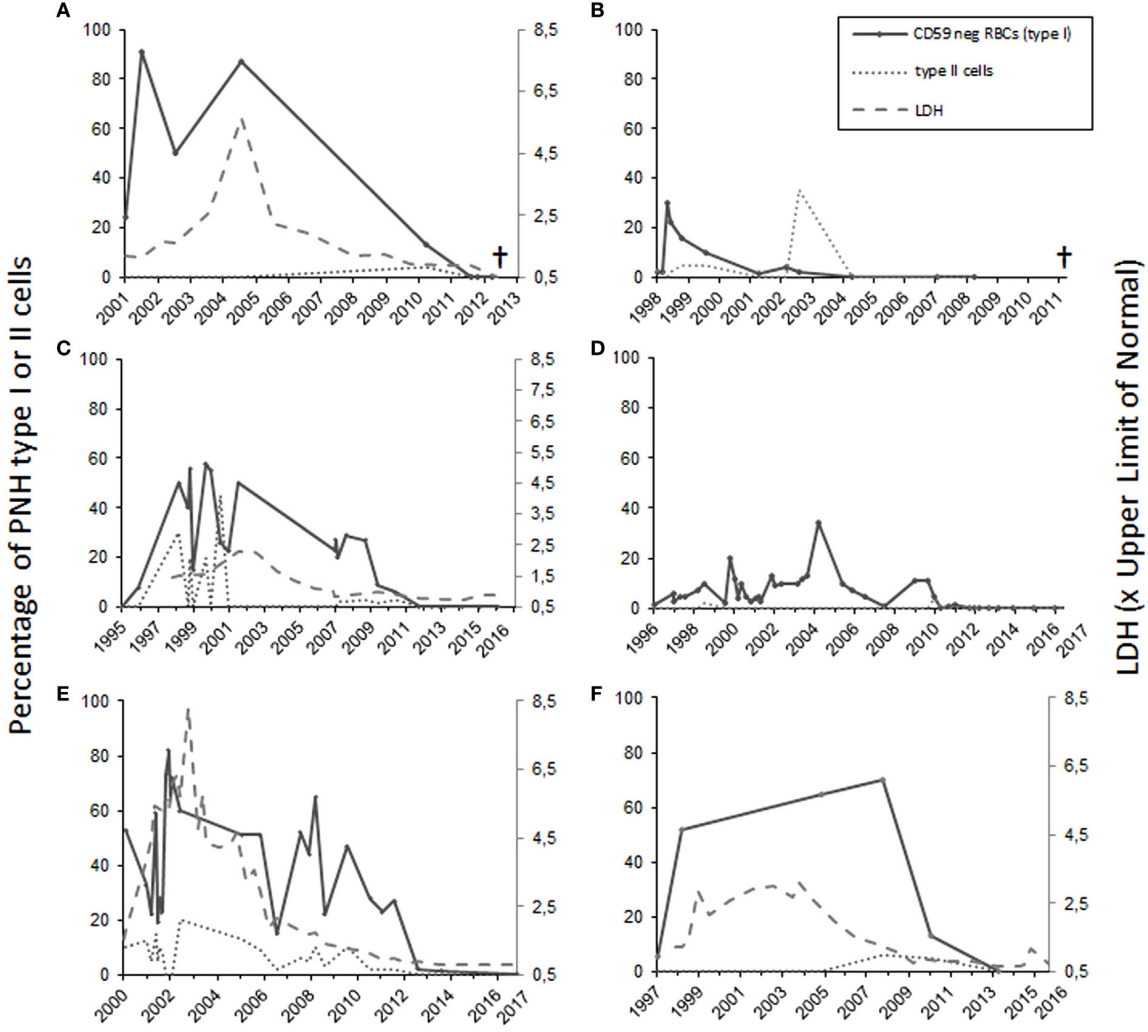
The percentage of totally and partially CD59-deficient red blood cells in patients 1–6 [**(A–F)**, respectively]. For patients 1, 3, 5, and 6 who had intravascular hemolysis, the lactate dehydrogenase levels (LD) are shown at right on the *Y*-axis as values in relation to the upper limit of normal **(A,C,E,F)**. Patients 1 and 2 **(A,B)** suffered from leukemia and subsequently died. Patients 3–6 are in remission.

Patient number 1 was diagnosed with AA in 1986 during a twin pregnancy. The patient had pre-eclampsia and a cesarean section was performed. The PNH diagnosis was made by Ham’s test in 1990 at the age of 23 years. The first flow cytometry test was performed in 2001 (24% CD59-negative erythrocytes) (Figure 1A). The patient was first treated with steroids without any positive outcome and with androgens for 2 years. In 1994, the patient was diagnosed with multiple sclerosis. In the mid 90s, the patient got several blood transfusions and twice more in 2005 and 2006. No thrombotic complications occurred. The patient was given anticoagulation treatment. Low molecular-weight heparin was used postoperatively after a gynecologic operation in 2006, and since 2008 the patient used aspirin. In 2012, erythrocytes, monocytes, and neutrophils were analyzed by the flow cytometry test, and no PNH clone was detected. The patient had no longer signs of hemolysis. In the same year, however, the patient was diagnosed with MDS and later with acute myeloid leukemia. The patient died of leukemia in 2013 at the age of 45 years.

Case 2 (female) was diagnosed with PNH in 1998 at the age of 56 years (30% CD59-negative erythrocytes by flow cytometry). At the time of diagnosis, the Hb level was 70 g/L, and the numbers of leukocytes 2.5 × 10^9^/L and platelets 6 × 10^9^/L. The patient had a tendency for bruising, petechiae, and bleeding on probing. In 1999, the patient’s BM sample showed hypoplastic features. The patient was treated with corticosteroids from 1998 until 2005. Androgen treatment was stopped because of elevated liver values. The CD59-negative erythrocyte clone peaked in 1998 after which it gradually declined. In 1999, the patient received cyclosporine treatment, which had a good effect on the anemia. In 2007, the patient recurrently developed anemia and thrombocytopenia. At that time, the RBCs had a normal CD59 expression level on flow cytometry (Figure 1B). The BM sample showed myelodysplastic changes and a systemic mastocytosis with c-*KIT* mutation was observed. In 2010, the patient was diagnosed with chronic myelomonocytic leukemia and azacytidine treatment was started. In 2011, the patient died because of acute ischemic heart disease during the leukemia treatment.

Case 3 (female) was diagnosed with AA in 1995 at the age of 21 years. At the time of diagnosis, the Hb level was 50 g/L, leukocytes 1.9 × 10^9^/L, and platelets 8 × 10^9^/L. The patient was treated with antithymocyte globulin, prednisolone, cyclosporine, and granulocyte growth factor. In 1996, 8% of the erythrocytes were CD59 deficient. When the flow cytometry was performed next time in 1998, 50% of the erythrocytes was type I CD59-deficient cells and 30% type II (partially CD59 deficient) cells (Figure 1C). No thrombotic complications or bleeding have occurred at any time of the disease history. The lactate dehydrogenase level was elevated earlier, but has been normal since 2006 (Figure 1C). The patient had two deliveries, in 2003 and 2007. During the first delivery, the patient was given blood transfusions and suffered from postpartum endometritis. The second delivery was without complications. In the 1990s, the patient had periods of hemoglobinuria. In 2012, the cyclosporine treatment was stopped, and only 0.2% of the erythrocytes were CD59 negative. Since 2013 the expression of CD59 has been normal on erythrocytes and the GPI-negative clone in neutrophils and monocytes has been 0.3–0.6% and the patient is symptom free.

Patient 4 (male) was diagnosed with AA in 1992, and the PNH diagnosis was made in 1998 when the patient was 25 years old (10% CD59-negative erythrocytes in flow cytometry). Before that the patient had had a small CD59-negative RBC clone (1.7–7%) since 1996. In 1992, the patient got several RBC and platelet transfusions. Cyclosporine and androgen treatment were started in 1992 with a good response leading to recovery from the cytopenias. The androgen treatment was discontinued in 2003 and cyclosporine treatment in 2012. Since then the patient has had no symptoms, and the laboratory parameters have been in the normal range. In 2010, CD59 expression on the erythrocytes was normal. In later controls, the expression has been either fully normal or the CD59-negative erythrocyte cell clone has been small (between 0.2 and 1.4%) (Figure 1D). The GPI-negative clone in neutrophils and monocytes has been in the range 0.2–1.5%.

Case 5 (male) was diagnosed with AA and PNH in 2000 at the age of 43 years. Fifty-three percent of the erythrocytes were totally CD59 deficient. In addition, 10% had a decreased expression (type II cells). In the BM sample, the cellularity was 25–30% of normal. At the time of diagnosis, the Hb level was 54 g/L, neutrophils 0.3 × 10^9^/L, and platelets 26 × 10^9^/L. The patient was initially treated with prednisolone for a short time. Later, the patient was given antithymocyte globulin treatment and cyclosporine for 1 year. Initially, the response to the immunosuppressive treatment was not sufficient, and an allogeneic stem cell transplantation with register donor was already being planned. In 2002, the transplantation was canceled because of a late response to the treatment. At that point, the Hb level was 122 g/L, neutrophils 0.77 × 10^9^/L, and platelets 96 × 10^9^/L. The patient had hemolysis and hemoglobinuria, fatigue, and erectile dysfunction. No thrombotic complications occurred. The anemia was compensated, and there was no transfusion dependency. Since 2009 the patient has been symptom free and the lab values stable, Hb level 155 g/L. The PNH clone has been declining (Figure 1E). In 2017, it was below 1% in both RBCs and neutrophils.

Patient 6 (male) was diagnosed with AA at the age of 43 years in 1997. At that time, the patient had pancytopenia and 5.4% of RBCs were CD59 deficient. The patient was treated with cyclosporine with a good response. In 1998, the size of the PNH clone was 52%. The patient had tendency for bruising, hemoglobinuria, and erectile dysfunction, but no thrombosis. Since 2009 the LD values normalized, and the patient did not report hemoglobinuria any more. In 2010, the size of the PNH clone had already clearly diminished, and in 2013, it was 0.3% in RBCs and 0.8% in neutrophils (Figure 1F).

Among all the six patients, the median time from the diagnosis until the remission was 12.5 years (range 6–15 years). For the two patients who developed leukemia, the diagnosis was made 26 and 12 years after the initial PNH/AA diagnosis, respectively. In one patient, the CD59-deficient cell clone disappeared 3 years earlier, and for the other one in the same year leukemia was diagnosed. For all six patients, the median CD59-deficient RBC clone size was 64% at highest (range 30–91%) (Figure 1). Four of the patients had classical PNH with signs of hemolysis. None of the patients received eculizumab therapy since it was not available at the time of diagnosis. All patients had underlying AA or another type of BM failure. Four of the patients were treated with cyclosporine, three with androgens, and five with corticosteroids. Two of the remission cases also got antithymocyte globulin, and one was given granulocyte growth factor. None of the patients was heavily transfusion dependent.

## Discussion

Paroxysmal nocturnal hemoglobinuria is a potentially serious illness with variable manifestations and outcomes. Reliably defined complete remissions from the disease have only rarely been described, and doubt exists whether a permanent cure from the disease can occur. In Finland, it has been possible to conduct a nation-wide study because it has a relatively small population (5.6 million) with a comprehensive public healthcare system, where rare cases are centralized to specialized healthcare. Thus, for this study, we were able to collect all patients diagnosed with PNH from a period of over 20 years, altogether 106 PNH patients. Indeed, among these we found six cases, where the PNH cell clones disappeared. In four cases, this was accompanied with spontaneous clinical remission, as well. By contrast, in two other cases—showing apparently the disappearance of the PNH clone—the disease developed into malignancy.

Our data show that spontaneous remissions are not as frequent as previously described, and the disappearance of the PNH cell clone can be related to dramatically different outcomes. In a Spanish patient cohort, spontaneous remission was reported to occur in as many as 30% of the patients (17/56), but the cases were not characterized further, and the spontaneous remission was not defined (8). We considered that the patients were in remission when they had no clinical signs of disease (i.e., no cytopenias, signs of hemolysis, or thrombosis) and no flow cytometry evidence for PNH. For the latter, a threshold level of 1.5% for GPI-anchor negative RBCs was employed, because levels lower than this may be artifacts due to staining or transportation. Small PNH-like clones may also be seen in healthy individuals (11). In a British material from the year 1995, spontaneous clinical remissions were reported in 15% of the patients (12/80) (7). The patients had a negative Ham’s test. In five cases, flow cytometry was later performed, and in all cases, the RBCs expressed normal levels of GPI-linked proteins. However, possible misdiagnoses with the earlier less specific Ham’s test cannot be excluded.

Nevertheless, as our study shows, true remissions occur, but the patients need to be carefully followed up for a potential emergence of malignancy. Among the six patients, where the PNH clone disappeared, four are healthy and have no treatment. The actual mechanisms or the reasons for the remissions are unknown. They may be spontaneous, have an immunological background, or be related to therapy. A proposed explanation has been that the PNH clone has a finite lifespan and that normal stem cells are capable of repopulating the BM (7). Recently, a case was described, where a decrease in the PNH clone did not restore normal hematopoiesis but instead was associated with clonal replacement (9). Notably, however, in this case, the PNH clone did not disappear but remained at a 15% level. All of our six patients had an underlying BM failure. Patients received treatments (e.g., cyclosporine, androgens, steroids, CSF, or antithymocyte globulin) that may also have affected the PNH cell clone or the cell clone could have lost its growth advantage (12) after repopulating the BM.

Paroxysmal nocturnal hemoglobinuria has previously been considered to be a monoclonal disorder, and the expansion of the GPI-deficient clone has been explained by extrinsic factors (13). Recent studies have shown that multiple clones are present, and a number of additional somatic mutations in the PNH clones have been detected (14, 15). The latter may lead to clonal selection. Partially, CD59-deficient type II cells are thought to emerge because of only a partially defective or absent PIG-A enzyme. As PNH is currently regarded as a multiclonal disease with clonal hierarchy (14) and a large number of cells are involved, a repairing mutation can not be the explanation for remission. In our view, the most likely possibility is that the immune system is eliminating cells through complement attack and/or by the formation of antibodies or by a cell-mediated immune response. If antibodies against cells develop, as may occur after blood transfusions, the clearance of CD59-negative cells could be further boosted. Cells lacking CD59 and CD55 would, after all, be more susceptible to complement-mediated clearance. Some antibodies may even be targeted against unique epitopes on GPI-anchor deficient cells.

Paroxysmal nocturnal hemoglobinuria is known to be a very variable disease, and this is also typical among the remission cases. The fact that the clone disappears may be related to different outcomes, total recovery, or BM suppression and development of leukemia. This provides a challenge to the clinician. Careful follow-up is needed to determine the direction to which the disease is progressing. Further studies on the mechanisms of spontaneous remissions could also provide clues for finding a curative treatment for PNH in the future.

## Ethics Statement

An ethical permission for the study was provided by the coordinating ethical committee at the Helsinki University Hospital Health Care District. A research permission was also obtained from the National Institute of Health and Welfare (THL, Helsinki, Finland), and the patients gave written informed consents for the study.

## Author Contributions

E-SK performed the research, analyzed data, and wrote the initial draft of the manuscript. HJ and SM designed and supervised the research, revised, and approved the manuscript. A-EA provided clinical information, reviewed, and approved the manuscript.

## Conflict of Interest Statement

E-SK and SM have received an investigator-initiated research grant and E-SK, A-EA, HJ, and SM have received speaker honoraria from Alexion Pharmaceuticals, Inc.
